# Is the risk of progressive multifocal leukoencephalopathy the real reason for natalizumab discontinuation in patients with multiple sclerosis?

**DOI:** 10.1371/journal.pone.0174858

**Published:** 2017-04-13

**Authors:** Julia Krämer, Jan-Gerd Tenberge, Ingo Kleiter, Wolfgang Gaissmaier, Tobias Ruck, Christoph Heesen, Sven G. Meuth

**Affiliations:** 1 Department of Neurology, Clinic of Neurology and Institute of Translational Neurology, Westfälische Wilhelms University, Münster, Germany; 2 Department of Neurology, St. Josef-Hospital, Ruhr-University-Bochum, Bochum, Germany; 3 Department of Psychology, University of Konstanz, Konstanz, Germany; 4 Department of Neurology, Institute of Neuroimmunology and MS (INIMS), University Medical Center Hamburg-Eppendorf, Hamburg, Germany; Julius-Maximilians-Universität Würzburg, GERMANY

## Abstract

**Background:**

Progressive multifocal leukoencephalopathy (PML) is one of the major risks of natalizumab therapy. Despite introduction of the currently employed PML risk stratification algorithm, the incidence of natalizumab-associated PML cases is not decreasing.

**Objectives:**

We addressed the following questions: How do natalizumab-treated multiple sclerosis patients and their treating physicians assess and deal with PML risk? Is PML risk the real reason for natalizumab discontinuation?

**Methods:**

699 natalizumab-treated multiple sclerosis patients and 99 physicians were included in this prospective observational study. Questionnaires were completed at 5 different time points. Patients were stratified into 5 subgroups according to the presence of PML risk factors (prior immunosuppression, anti-JCV antibody status, treatment duration). Patients with prior immunosuppression (n = 30, treated by n = 7 physicians) were excluded from analyses, because patient numbers were too small. Patients’ anti-JCV antibody index was not considered because data recruitment ended in 2014. Using Bayesian network and regression analysis, we examined the relationship between different patient- and physician-related factors and patients’ discontinuation of natalizumab.

**Results:**

Patients of all subgroups and physicians assessed the PML risk as low. Overall patient adherence to natalizumab was high (87%). Only 13% of patients discontinued therapy. Natalizumab treatment cessation was associated with different patient- and physician-related factors (physicians’ assessment of general PML risk, number of treated patients per year, natalizumab treatment duration, relapses during the course of study) upon which only physicians’ judgment on treatment continuation, patients’ perception of personal PML risk, and JCV seroconversion showed significant relationships.

**C*onclusion*:**

According to the currently employed risk stratification algorithm, the *objective* PML risk probably doesn’t play a dominant role in a patients’ decision to continue or stop natalizumab treatment. The decision-making process is rather guided by *subjective* views and experiences of patients and treating neurologists. Treating physicians should consider this discrepancy in their advice to improve the risk-benefit-ratio for the individual patient.

## Introduction

Natalizumab (NTZ) (Tysabri; Biogen, Cambrige, MA, USA), a humanized recombinant monoclonal antibody that inhibits leukocyte extravasation into the central nervous system by targeting VLA-4, was approved for the treatment of active relapsing-remitting multiple sclerosis (RRMS) in the US in 2004 on the basis of interim analyses of two large phase III trials (AFFIRM and SENTINEL) [1,2]. NTZ was, however, voluntarily withdrawn in 2005 due to its association with the occurrence of progressive multifocal leukoencephalopathy (PML). PML is a rare but severe opportunistic infection of the central nervous system that is caused by lytic infection of oligodendrocytes and astrocytes by the ubiquitous John Cunningham virus (JCV). PML leads to death in 20% of affected patients or serious disability in 40% of survivors [3–5]. Nevertheless, a risk-benefit review after establishment of an intensive global risk management program resulted in the reintroduction of NTZ in 2006 (reintroduced in the US and first approved in the EU). To date, more than 664 cases of PML in 152.500 NTZ-treated patients with RRMS have been reported (6 June 2016) [6]. Based on these data, the overall PML risk in natalizumab-treated patients is currently estimated at 4.22/1000 [6]. Also described as the “dark side of immunotherapy” [7], PML therefore remains the major risk limiting the use of NTZ in patients with RRMS. Three independent factors identified in all NTZ-treated patients with PML are established biomarkers for PML risk and are currently employed for PML risk stratification: the duration of NTZ treatment, especially after 24 months, prior use of immunosuppressants (IMS +), and positive serostatus for anti-JCV antibodies (JCV Ab +) [8]. However, this three-stage risk stratification algorithm does not allow for a precise prediction of PML risk in individual patients [4,9]. Moreover, its usefulness for risk stratification was regarded to be limited because of the imbalance between the high percentage of patients that are seropositive for JCV, and the rare occurrence of PML [10]. To improve the safety and efficacy of NTZ, and to guide individually tailored clinical decisions, immunological markers such as CD49d, CD11a, and CD62L expression on leukocytes, lipid-specific oligoclonal immunoglobulin M bands in cerebrospinal fluid, the intrathecal immunoglobulin G index, JCV-specific activated T effector memory cells, and genetic screening approaches have been proposed [9–13]. Lower body weight, aging, and immunosenescence were also considered to be risk factors for PML [14,15]. Recently, it was demonstrated that immunosuppression-naïve, JCV Ab + NTZ-treated MS patients with a low anti-JCV Ab index carry a several-fold lower PML risk than those with a high index, and the quantification of anti-JCV Ab levels was suggested to improve PML risk stratification [16,17]. In March 2016, the anti-JCV Ab index testing every six months in immunosuppression-naïve, JCV Ab + patients with a low anti-JCV Ab index (≤ 0.9) and NTZ treatment > 24 months was recommended by Biogen according to the European Medicines Agency/ Paul-Ehrlich-Institute. Depending on the anti-JCV Ab index and the NTZ treatment duration, the PML risk is estimated to be 1/10000–1/100 [18,19]. In anti-JCV antibody seronegative (JCV Ab -) patients, the risk for PML is considered to be negligible (1/10000) [3,16,18], irrespective of the treatment duration. Due to the lack of available data in patients with ≥ 6 years of treatment exposure, the PML risk estimate is limited beyond 6 years of treatment [6]. Though, despite advances in risk stratification, the PML incidence in NTZ-treated patients with RRMS has not declined over the last few years [10,20]. This might be due to the fact that patients’ and physicians’ *subjective* risk estimations and acceptance differ from the *objective* PML risk, and that factors other than the *real* PML risk according to the common used PML stratification algorithm decide on continuation or cessation of NTZ therapy.

The current study aims to address the following questions which are of high relevance for daily practice: *I*. How do NTZ-treated RRMS patients with different PML risk levels and their treating physicians assess and deal with the PML risk in clinical routine? *II*. Is the decision to continue or stop NTZ only taken on the basis of the *objective* PML risk according to the three-stage risk stratification algorithm [8], or do other patient- and physician-related factors also play a major role?

## Materials and methods

### Study design

The present study was part of the prospective, multicenter, non-interventional, observational program PERCEPT (PERCEPTion of risk in patients on NTZ and physicians) performed in Germany between November 2011 and August 2014. The participants were RRMS patients treated with NTZ, and their treating neurologists in clinical routine. Physicians were asked to include all consecutive patients already treated with NTZ and those for whom NTZ therapy was planned. Inclusion criteria for patients were: age ≥ 18 years, diagnosis of RRMS, current or planned treatment with NTZ according to current German guidelines [21], and written informed consent. Exclusion criteria corresponded to the contraindications listed in the product information leaflet of NTZ [22]. Study data were collected at 5 time points: at study entry (= visit 1) and 1, 2, 6, and 12 months later (= follow-up visits 2–5). All visits followed a specific schedule (S1 Fig). At each visit, physicians had to document in an electronic case report form and were required to complete an online questionnaire. Additionally at visit 2, physicians had to complete a decisional conflict scale investigating decision conflicts concerning the NTZ therapy. Patients were asked to complete a questionnaire on each visit, and a decisional conflict scale on visit 2 to 5.

### Participants

Of 801 patients participating in PERCEPT, we included 699 patients (489 female, mean age 38.5 ± 9.98 years) who were ≥ 18 years old and for whom the following information were known at baseline and plausible: duration of MS disease, number of previous NTZ infusions, anti-JCV antibody serostatus, date of birth, date of baseline. According to the PML risk factors used in the common three-stage risk stratification algorithm (IMS +, JCV Ab +, and duration of NTZ treatment longer than 24 months (NTZ > 24 months)) [8], patients were divided into five subgroups: group A: JCV Ab–; group B: JCV Ab +, IMS -, NTZ ≤ 24 months; group C: JCV Ab +, IMS -, NTZ > 24 months; group D: JCV Ab +, IMS +, NTZ ≤ 24 months; group E: JCV Ab +, IMS +, NTZ > 24 months. Quantification of anti-JCV Ab index was not applied because data recruitment ended in 2014. The number of patients in subgroups D and E was small (9 and 21 respectively) and made comparison with the larger groups A-C difficult, potentially affecting the validity of the results. We therefore decided to conduct all statistical analyses only with subgroups A-C (669 patients in total).

A total of 99 neurologists from 73 multiple sclerosis (MS) treatment centers spread all over Germany and arbitrarily selected participated in PERCEPT. All centers were experienced in treating MS patients and comprised hospitals and office-based medical practices. We included those 92 physicians who treated the 669 selected patients in subgroups A-C.

### Questionnaires

Questionnaires for physicians and patients used multiple-choice formats, polar questions (yes–no questions) or visual analog scales (VAS) for subjective ratings.

Patients were asked for their age, gender, year of manifestation of first symptoms, year of diagnosis, previous MS therapies, previous immunosuppressive therapies, relapses in the year before inclusion and before starting NTZ, the date of starting NTZ, and the number of NTZ infusions. Physicians were asked for their age, gender, work place, self-classified MS expertise (from low to excellent, 4-point Likert scale), number of patients treated per year, and for the reason of patients’ NTZ treatment discontinuation (1 = ‘too high therapeutic risk’, 2 = ‘patients’ desire to change therapy’, 3 = ‘withdrawn consent’, 4 = ‘lack of efficacy’, 5 = ‘serious adverse events’, 6 = ‘pregnancy’, 7 = ‘other’). With the latter question, several answers were possible. Moreover, they were asked if the patient received regularly (= every four weeks) or irregularly NTZ infusions, or if NTZ was interrupted for a longer time period. At visit 2–5, physicians were asked if relapses, adverse events, serious adverse events or pregnancy occurred since the last visit, or if JCV Ab testing was performed in the meantime. If JCV Ab testing was conducted, physicians had to indicate the date, the result, and the type of JCV Ab testing that was used. Adverse and serious adverse events were reported according to applicable regulations of the German drug authorities. Physicians had to assess the expanded disability status scale (EDSS) score at each visit. Moreover at baseline, they were requested to specify the EDSS before starting NTZ. Physicians and patients were asked to rate the severity of MS in general on a VAS (1 = ‘rather benign’, 25 = ‘rather severe’). Physicians were additionally asked to evaluate the severity of MS in the individual NTZ-treated patient. Physicians and patients were requested to value the efficacy of NTZ (a multiple-choice question from ‘a lot’ to ‘not known’, 5-point Likert scale, was used for physicians and a polar question for patients). All participants had to assess the PML risk in general on a VAS (1 = ‘low’, 25 = ‘high’) and patients their personal PML risk compared to other patients in the same situation on a VAS (1 = ‘lower’, 25 = ‘higher’). Moreover, physicians had to judge if they would continue or discontinue the NTZ therapy in the individual patient (VAS; 1 = ‘more likely to continue’, 25 = ‘more likely to discontinue’). Patients were asked polar questions on whether the risk of NTZ (PML) makes them afraid and if they are prepared to take therapeutic risks to live a “normal” life. Patients were requested to evaluate their actual status of health at baseline compared to a year ago, and on each subsequent visit compared to prior visits (1 = ‘considerably worse’, 2 = ‘noticeably worse’, 3 = ‘minimally worse’, 4 = ‘no change’, 5 = ‘minimally improved’, 6 = ‘noticeably improved’, 7 = ‘considerably improved’, 7-point Likert scale). Moreover, patients were asked to express their satisfaction with their quality of life (QoL) on a 5-point Likert scale (1 = ‘not at all’, 2 = ‘somewhat’, 3 = ‘moderately’, 4 = ‘quite’, 5 = ‘very’). We considered patients to be satisfied with their QoL if they selected the specifications ‘somewhat’, ‘moderately’, ‘quite’ or ‘very’. Patients were asked to assess a potential wheelchair dependency on a VAS (1 = ‘It wouldn’t matter much’, 25 = ‘It’s the worst thing I can imagine’). An English and German version of physicians’ and patients’ questionnaires are given in S1 Appendix and S2 Appendix.

### Ethical approval

PERCEPT was approved by the ethics committee of the Hamburg Chamber of Physicians (PV3983 10.4.2012 and PV3955 5.1.2012).

### Statistical analyses

The prechosen significance level for all confirmatory tests was set to α = 0.05. Differences of results between patients who stopped (Patients_Stopped NTZ_) and who continued the NTZ therapy during the observation period (Patients_Cont. NTZ_) were analyzed by two-sided t-tests for independent samples. All tests were Bonferroni corrected for multiple comparisons.

We conducted a Bayesian network analysis with the bnlearn R package to examine possible relationships between patient- and physician-related factors and patients’ discontinuation of NTZ therapy. For details of this method see [23]. We investigated the following patient-related factors: duration of NTZ treatment until study end or treatment discontinuation (> / ≤ 24 months), result of the anti-JCV antibody test at baseline (positive/ negative) and before the end of study/NTZ treatment (positive/ negative), JCV seroconversion during observation period (yes/ no), relapses during the course of study (0/ ≥ 1 relapse), patients’ evaluation of efficacy of NTZ, of severity of MS, of personal and general PML risk, of fear of PML, and of willingness to take therapeutic risks. Concerning the patients’ evaluation of different factors, we always used their last answer given before the end of study/NTZ treatment discontinuation. We investigated the following physician-related factors: number of treated MS-patients per year, type of treatment center, physicians’ assessment of PML risk in general (mean value over all visits), and judgment on continuation/discontinuation of NTZ in the individual patient (last value specified before end of study/NTZ treatment discontinuation). Due to physicians’ low response rate to the questions regarding assessment of efficacy of NTZ, severity of MS, and self-classified MS expertise, we did not include those physician-related factors in the Bayesian network analysis. The Bayesian network analysis was based on information of 479 patients of the subgroups A-C and the associated 92 neurologists with complete data on the above-mentioned patient- and physician-related factors. The network was fitted using the hill-climb algorithm with Akaike information criterions score (restart = 2000, perturb = 5). Answers based on the VAS were turned into categorical data by splitting into a 50%-range (0–12.5 points and 12.6–25 points).

In addition, we conducted a regression analysis in an exploratory way to analyze the relationships between NTZ discontinuation and those factors that were found by the Bayesian network analysis to be significantly associated with patients’ decision to stop NTZ therapy in the whole sample.

## Results

### Main demographic and professional features of participating neurologists at baseline

The majority of neurologists was male (70%) and the mean age was 47.7 years (standard deviation (SD) = 10.2 years). 72% were working in office based medical practices, 27% in hospitals, and 1% in both. 94% evaluated their MS expertise as excellent or high. 51% of physicians treated ≤ 250 MS patients per year, 29% 251–500 patients per year, and only 3% more than 1000 patients per year. Descriptive details of participating neurologists are given in Table 1.

**Table 1:** Descriptive demographic and professional details of participating neurologists at baseline. N describes the number of neurologists with fully completed questionnaires at baseline.

		Absolute numbers	Relative numbers in percent
**Gender** (N = 92)	Male	63	68,5
	Female	27	29,3
	Unknown	2	2,2
**Age** (N = 78)	Mean	47,7	
	Min.	26	
	Max.	67	
	Standard deviation	10,2	
**Self-classified MS expertise** (N = 92)	Excellent	40	43,5
	High	40	43,5
	Moderate	5	5,4
	Low	0	0
	Unknown	7	7,6
**MS-Patients treated per year** (N = 92)	0–250	47	51,1
	251–500	27	29,3
	501–1000	15	16,3
	>1000	3	3,3
**Type of treatment center** (N = 92)	Office-based medical practice	65	70,7
	Hospital	24	26,1
	Office-based medical practice and hospital	1	1,1
	Unknown	2	2,2

### Observational period and main demographic and disease-related features of participating patients at baseline

The average observation period was 264.8 ± 125.5 days. Group A was the largest group (374 patients). Groups B (162) and C (133) were significantly smaller. Most patients of all subgroups were female (group A: 70%, group B: 71%, group C: 68%). Patients of group C were older, had the longest disease duration (DD), and had received the most NTZ infusions at baseline compared to those of subgroups A and B. Descriptive details of patients of all subgroups are given in Table 2 and Table 3. 69 patients were treatment naive at inclusion, while the majority of 600 patients had already received approved disease modifying therapies (DMT) (576), immunoglobulins (8), plasmapheresis (1), steroid pulse therapy (1) or immunosuppressants (14; mitoxantrone and azathioprine) as last therapy before starting NTZ (see S1 Table).

**Table 2:** Descriptive demographic data of patients of the subgroups A-E at baseline. ELISA = enzyme-linked immunosorbent assays; IMS + /- = prior / no prior use of immunosuppressants; JCV Ab + /- = positive / negative serostatus for anti-JCV antibodies; NTZ = natalizumab; NTZ > / ≤ 24 months = NTZ treatment longer / less than 24 months.

	Total number of patients	Gender		Type of used JCV antibody testing			
		Male	Female	STRATIFY JCV	ELISA other than STRATIFY JCV	unknown or externally determined	cerebrospinal fluid examination
**Group A** (JCV Ab -)	374	112	262	354	6	3	1
**Group B** (JCV Ab +, IMS -, NTZ ≤ 24 months)	162	47	115	152	2	1	0
**Group C** (JCV Ab +, IMS -, NTZ > 24 months)	133	42	91	130	2	0	0
**Group D** (JCV Ab +, IMS +, NTZ ≤ 24 months)	9	0	9	9	0	0	0
**Group E** (JCV Ab +, IMS +, NTZ > 24 months)	21	9	12	20	1	0	0

**Table 3:** Disease-related data of patients of the groups A-E at baseline. ARR = annualized relapse rate; EDSS = Expanded Disability Status Scale; IMS + /- = prior / no prior use of immunosuppressants; JCV Ab + /- = positive / negative serostatus for anti-JCV antibodies; NTZ > / ≤ 24 months = natalizumab treatment longer / less than 24 months.

		EDSS at baseline	EDSS before starting NTZ	ARR before inclusion	ARR before starting NTZ	Age/years	Disease duration/years	Duration of NTZ therapy/ months	Number of NTZ infusions
**Group A** (JCV Ab -)	Mean	3,05	3,15	1,08	2,16	36,5	8,3	21,4	19,7
	Median	3	3	1	2	36	6,98	11,3	11
	Min.	0	0	0	0	18	0,08	0	0
	Max.	8	8,5	7	7	67	32,2	73,3	77
	SD	1,61	1,55	1,32	1,32	9,21	5,87	22,2	20,6
**Group B** (JCV Ab +, IMS -, NTZ ≤ 24 months)	Mean	2,94	3,16	1,47	2,19	38,8	6,63	7,05	7,01
	Median	3	3	1	2	39	5,22	4,75	5
	Min.	0	0	0	0	18	0,06	0	0
	Max.	6,5	6,5	6	12	70	25,9	23,9	26
	SD	1,47	1,47	1,41	1,46	10,5	5,52	7,14	7,05
**Group C** (JCV Ab +, IMS -, NTZ > 24 months)	Mean	3,12	3,25	0,3	2,41	42,9	11,5	51,7	46,4
	Median	3	3	0	2	44	10,2	54,3	47
	Min.	0	0	0	0	22	3,42	26,5	1
	Max.	7	7	4	10	70	32	77,7	77
	SD	1,46	1,35	0,67	1,53	9,47	5,65	13,2	13,6
**Group D** (JCV Ab +, IMS +, NTZ ≤ 24 months)	Mean	4	4,44	1,11	2,22	41	15,5	9,45	9,11
	Median	3,5	5	1	2	40	14,4	8,03	9
	Min.	1,5	1,5	0	1	29	8,6	0	1
	Max.	6,5	6,5	5	5	55	25,5	23,9	19
	SD	1,68	1,72	1,62	1,3	8,9	5,18	8,54	7,62
**Group E** (JCV Ab +, IMS +, NTZ > 24months)	Mean	4,64	4,5	0,24	2,62	43,7	15,3	49,4	46,1
	Median	4	4,5	0	2	44	15,1	49,5	44
	Min.	2	1,5	0	0	23	7,9	25	26
	Max.	7,5	6,5	2	6	66	24,4	70,5	75
	SD	1,63	1,49	0,54	1,56	11,8	4,98	14,6	13,9

### Adherence to natalizumab treatment

Most patients received NTZ infusions regularly every four weeks independently of PML risk (82% (529/644*) at visit 1, 90% (587/649*) at visit 2, 91% (555/607*) at visit 3, 89% (512/573*) at visit 4, and 82% (359/436*) at visit 5) (* indicates the number of patients with answered question). 18.8% (group A: 64/374; group B: 33/162; group C: 29/133) of all patients received NTZ infusions “irregularly”, mostly every 5, 6 or 7 weeks, according to an alternative therapy regimen (prolonged infusion interval). In 13.2% (group A: 47/374; group B: 10/162; group C: 31/133) of all patients NTZ was interrupted for a longer time period. The most common reasons for alternative therapy regimens were infections, professional or private reasons and for longer interruptions patients’ desire, planned or accidental pregnancies, PML risk, other diseases than MS, and adverse events (AE).

### Reasons for discontinuation of natalizumab treatment

13% (90/669) of all included patients discontinued NTZ during the observation period with the highest rate of discontinuations in group B (group A: 42/374 (11.2%) patients; group B: 33/162 (20.4%) patients; group C: 15/133 (11.3%) patients). Physicians were asked for the reason why patients’ treatment was discontinued. The main reasons given for treatment discontinuation of patients of all subgroups were high therapeutic risk (33/90) (group A: 14/42 (33%); group B: 10/33 (30%); group C 9/15 (60%)), and at a similar percentage, the patients’ desire to change therapy (37/90) (group A: 15/42 (36%); group B: 14/33 (42%); group C 8/15 (53%)). However, for patients of the groups A and B, other reasons such as lack of efficacy (group A: n = 5; group B: n = 6), adverse events (AEs) (group A: n = 4), serious adverse events (SAE) (group A: n = 3; group B: n = 1), the wish to become pregnant (group A: n = 1), and pregnancy (group A: n = 2; group B: n = 2; group C: n = 1) were also provided. A total of 46 SAEs were documented for 28 patients throughout the study period (S2 Table). Most SAEs were MS relapses leading to hospitalization. A relationship between SAEs and NTZ treatment was suspected in 11 cases. 3 patients (group B: 1; group C: 2) developed a PML. Two of them (group C) suffered of immune reconstitution inflammatory syndrome after NTZ discontinuation. If courses of plasmapheresis were performed in those patients is not known. One pregnancy was a live birth without defects and one resulted in spontaneous abortion. Information about the outcome of the other 3 pregnancies was not available.

13% (50/374) of JCV Ab negative patients showed JCV seroconversion during the observation period and were thereby switched to group B (28) or C (22) depending on the duration of NTZ treatment. Of those, 26% (13/50) stopped NTZ treatment, mostly due to high therapy risk (total 10/13; 67% (6/9) of patients who switched from group A to B and all patients (100%, 4/4) who switched from group A to C during the observation period). 20 patients who initially belonged to group B at baseline became part of group C during the course of the study due to a NTZ treatment duration > 24 months. Of those, only one stopped NTZ to change therapy.

### MS disease activity, status of health, and quality of life

Under NTZ treatment, the EDSS score remained stable or improved in 77% of all patients (515/669; Patients_Stopped NTZ_ = 72%, 65/90; Patients_Cont. NTZ_ = 78%, 450/579) and most patients were free from relapses (87%, 583/699; Patients_Stopped NTZ_ = 72.2%, 65/90; Patients_Cont. NTZ_ = 89.5%, 518/579). However, in Patients_Stopped NTZ,_ the mean total number of relapses per patient was higher compared 0to Patients_Cont. NTZ_ (Patients_Stopped NTZ_ = 0.48 ± 0.95 points; Patients_Cont. NTZ_ = 0.13 ± 0.43 points; p < 0.0001). Both Patients_Stopped NTZ_ and Patients_Cont. NTZ_ evaluated MS in general as a serious disease (Patients_Stopped NTZ_ = 19.1 ± 4.5 points; Patients_Cont. NTZ_ = 18.3 ± 4.5 points) and the threat of wheelchair dependency (Patients_Stopped NTZ_ = 19.73 ± 5.37 points; Patients_Cont. NTZ_ = 19.84 ± 5.05 points) as substantial. The majority of Patients_Stopped NTZ_ and Patients_Cont. NTZ_ assessed their actual status of health as equal as or better than before (Patients_Stopped NTZ_ = 61.1%, 55/90; Patients_Cont. NTZ_ = 69.1%, 400/579). Both Patients_Cont. NTZ_ and Patients_Stopped NTZ_ showed a slight upward trend in their evaluation of actual status of health during the first three visits. While the assessment of actual status of health further showed a slight improvement in Patients_Cont. NTZ_, it worsened at the end of the study in Patients_Stopped NTZ_ (S2 Fig). The majority of Patients_Stopped NTZ_ and Patients_Cont. NTZ_ assessed their QoL as satisfactory (Patients_Stopped NTZ_: 94,4%, 85/90; Patients_Cont. NTZ_: 95.8%, 555/579).

### Patients’ and physicians’ perception of PML risk

**Fig 1:**
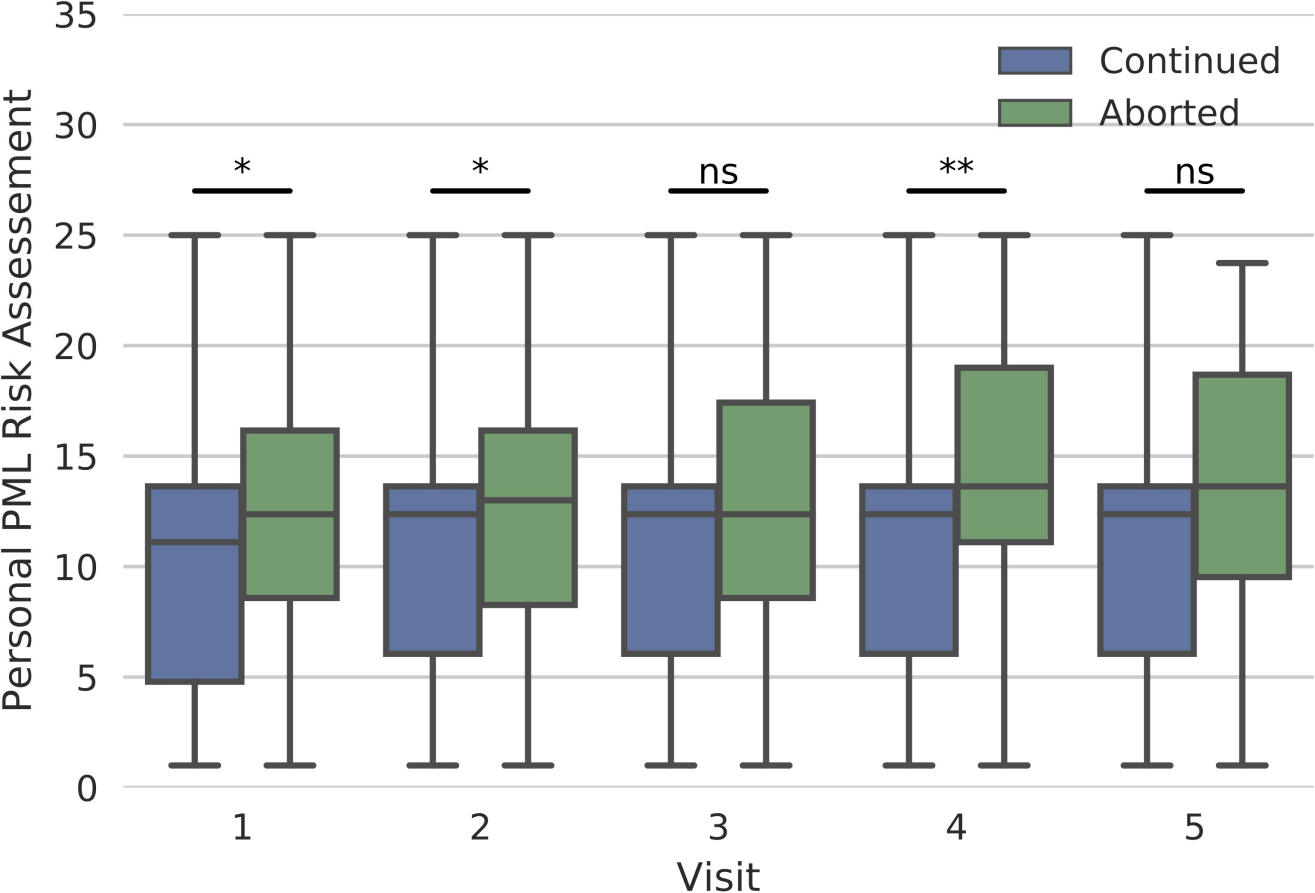
Assessment of personal PML risk at visit 1 to 5 for patients continuing and discontinuing natalizumab (NTZ). Patients discontinuing NTZ (green) assessed the personal PML risk higher on a visual analog scale from 0 to 25 points (0 = ‘low’, 25 = ‘high’) in all visits than patients continuing NTZ (blue) (*p < 0.01; **p < 0.0002; ns = not significant).

Patients of all subgroups showed on average comparable low risk estimations of general (PML_General_) and personal PML risk (PML_Personal_). Both risk estimates correlated moderately with each other (*p* < 0.001; r = 0.5). When analyzing isolated Patients_Cont. NTZ_ and Patients_Stopped NTZ_, both groups assessed the PML_Personal_ on average at a similarly low level on a VAS from 0 to 25 points (PML_Personal_ of Patients_Stopped NTZ_: 13.0 ± 4.8 points, 84/90 statements; PML_Personal_ of Patients_Cont. NTZ_: 10.8 ± 4.8 points, 563/579 statements). However, Patients_stopped NTZ_ assessed the PML_Personal_ higher in all visits than Patients_Cont. NTZ_ (Fig 1). For the exact values of perceived personal PML risk from Patients_Cont. NTZ_ and Patients_stopped NTZ_ of all subgroups, see S3 Table.

**Fig 2:**
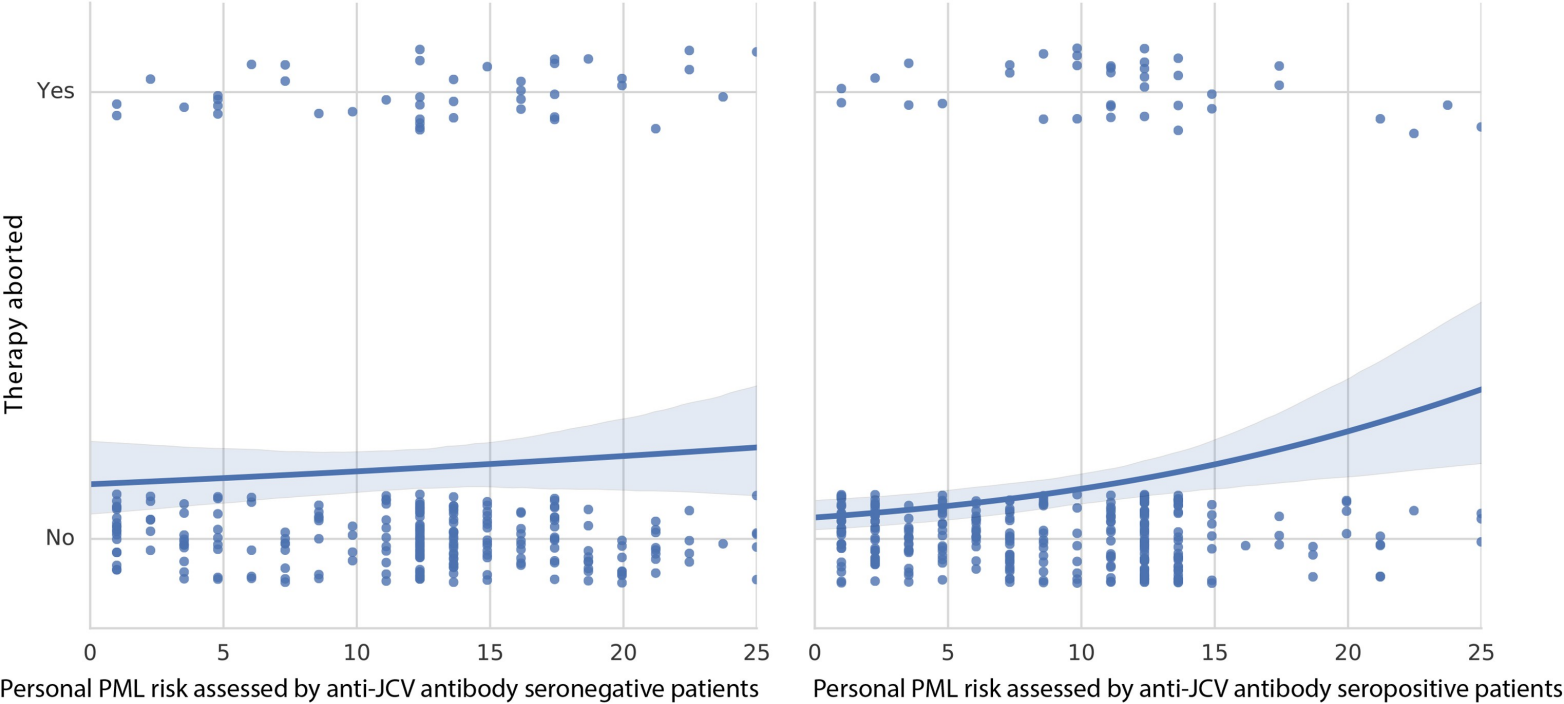
Relationship between patients’ assessment of personal PML risk and the proportion of patients stopping therapy. Patients’ assessment of personal PML risk on a visual analog scale from 0 to 25 points (0 = ‘low’, 25 = ‘high’) did not correlate with the proportion of patients stopping therapy in JCV Ab–patients (left side) and correlated only weakly in JCV Ab + patients (right side).

Patients’ assessment of personal PML risk did not correlate with the proportion of patients stopping NTZ therapy in JCV Ab–patients and correlated only weakly in JCV Ab + patients (Fig 2).

**Fig 3:**
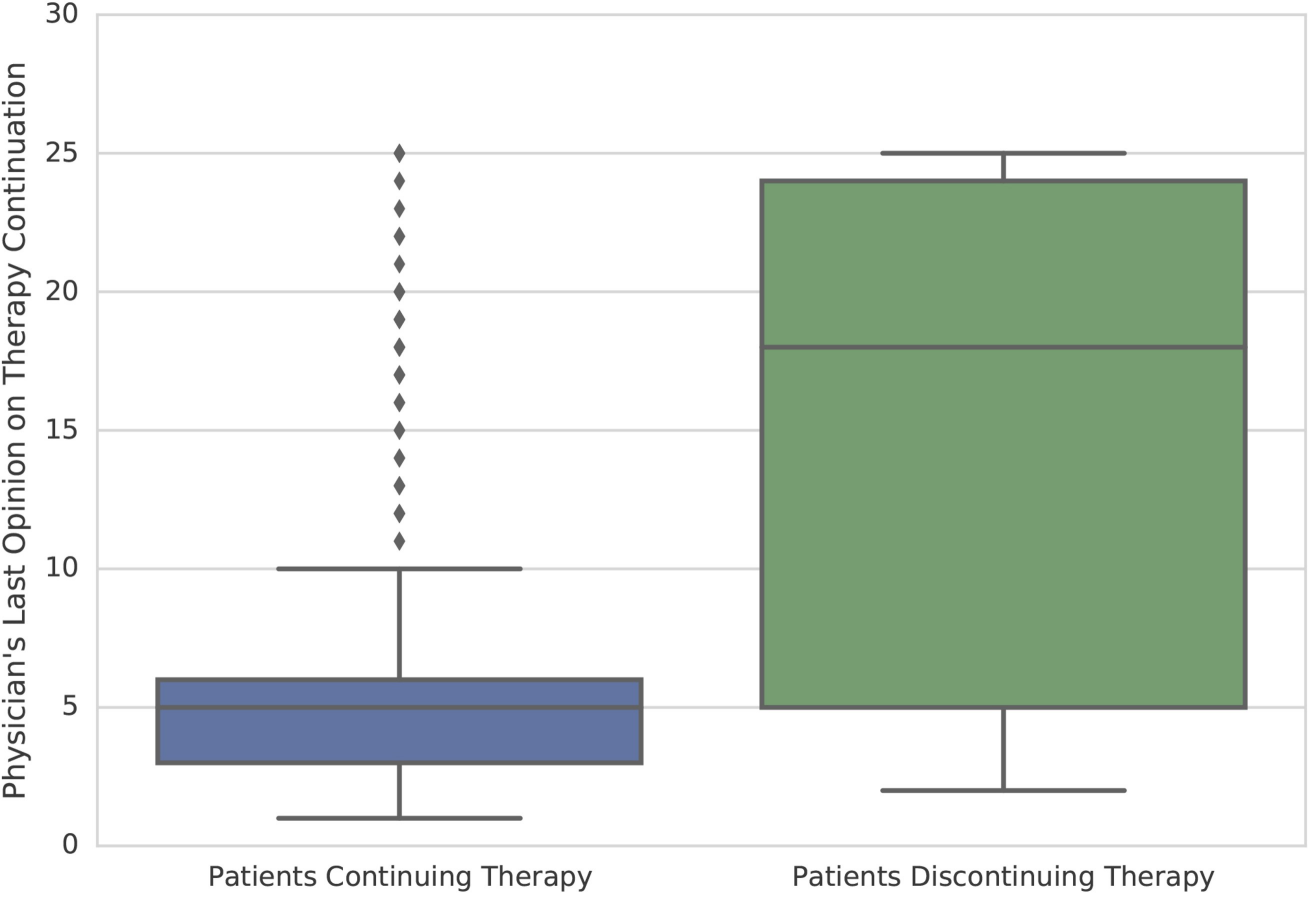
Significant relationship between physicians’ last statement on continuation or discontinuation of natalizumab and the proportion of patients continuing or stopping therapy. Physicians’ last judgment on continuation of therapy on a visual analog scale from 0 to 25 points (1 = ‘rather continue natalizumab’, 25 = ‘rather discontinue natalizumab’) had an influence on the proportion of patients continuing/discontinuing natalizumab.

Physicians also assessed the PML risk in general as low and mainly voted for continuation of treatment (S4 Table). The physicians’ last judgment on continuation of therapy (before end of study/NTZ treatment discontinuation) had an influence on the proportion of patients continuing/discontinuing NZT (Fig 3).

### Factors influencing patients’ decision to stop NTZ treatment

**Fig 4:**
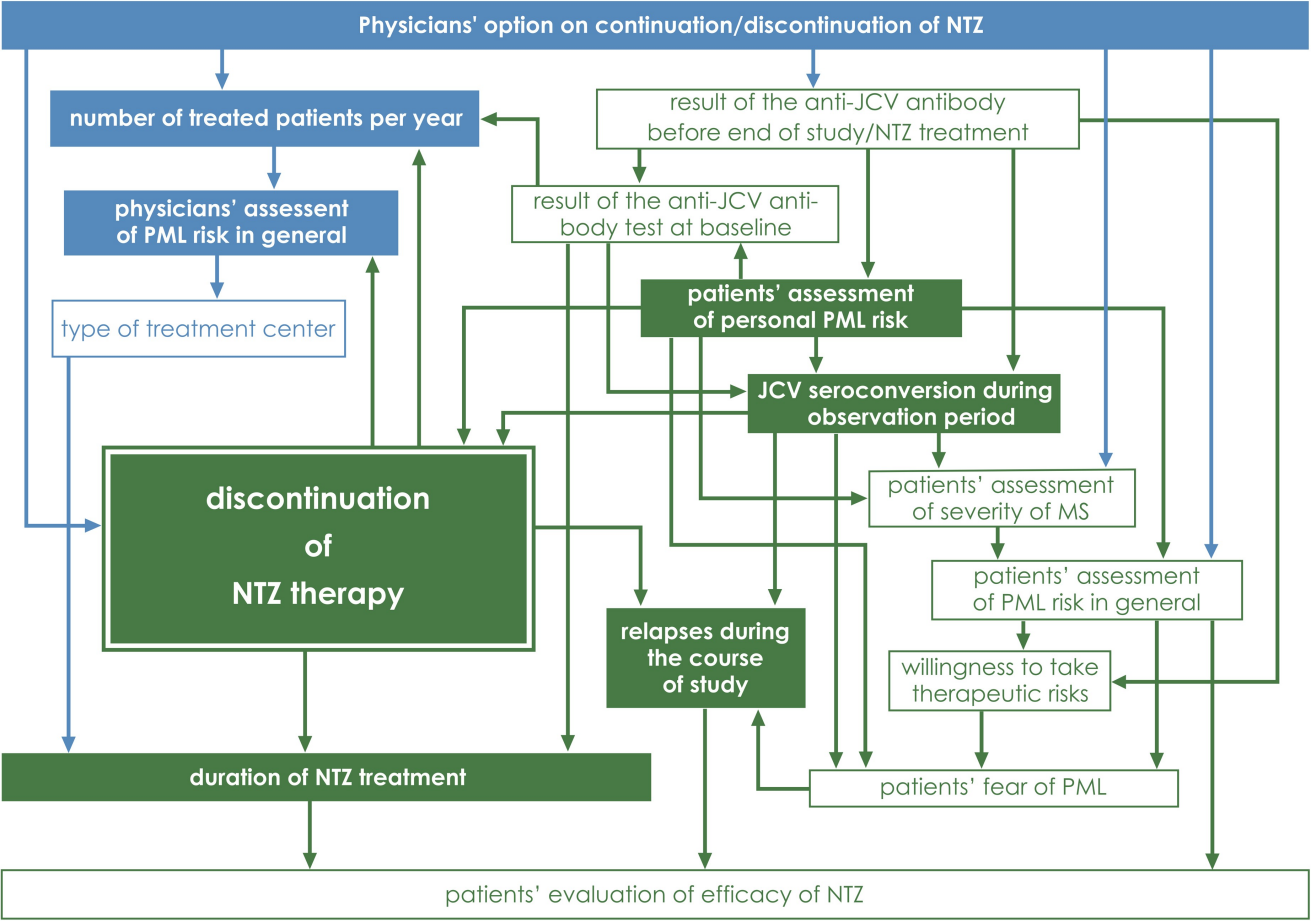
Bayesian network learned from the information of patients of the subgroups A-C and associated neurologists with the bnlearn R package. All patient-related factors are colored in green and all physician-related factors are colored in blue. JCV seroconversion during observation period, physicians’ opinion on continuation/discontinuation of NTZ in the individual patient, assessment of PML risk in general, number of treated patients per year and patients’ perception of personal PML risk, duration of NTZ treatment and relapses during the course of the study were directly associated with patients’ discontinuation of NTZ therapy (those factors are pictured as completely colour-filled boxes). For all other examined factors we found no relationship with NTZ treatment cessation (those factors are pictured as white boxes).

Using Bayesian network analysis, we could identify 7 factors which were associated with patients’ decision to stop NTZ therapy: JCV seroconversion during observation period, physicians’ judgment on continuation/discontinuation of NTZ in the individual patient, physicians’ assessment of PML risk in general, physicians’ number of treated patients per year, patients’ perception of personal PML risk, duration of NTZ treatment (> / ≤ 24 months), and if patients had relapses during the course of the study or not (colour-filled boxes in Fig 4). For all other examined factors we found no relationship with NTZ treatment cessation (white boxes in Fig 4). The result of the anti-JCV antibody test at baseline was related to the result of the last anti-JCV antibody test performed before end of study/treatment discontinuation and both to JCV seroconversion. As assumed, physicians’ judgment on continuation/discontinuation of NTZ, assessment of PML risk in general, number of patients treated per year, and type of treatment center were related to each other (blue factors in Fig 4). Patients’ assessment of personal and general PML risk, of MS severity, of efficacy of NTZ, patients’ fear of PML, willingness to take therapeutic risks, and relapses during the course of study were also related to each other (green factors in Fig 4). The result of the anti-JCV antibody test at baseline, of the last anti-JCV antibody test performed before end of study/treatment discontinuation, and JCV seroconversion during study were associated with patients’ assessment of personal PML risk. Interestingly, we found a relationship between physicians’ number of patients treated per year and their perception of PML risk in general and patients’ fear of PML and willingness to take therapeutic risks. Moreover, the occurrence of relapses during the course of the study had an influence on patients’ evaluation of efficacy of NTZ.

Regression analysis revealed that only patients’ perception of personal PML risk, physicians’ judgment on continuation/discontinuation of NTZ, and the fact if patients showed JCV seroconversion during study or not were significantly related to NTZ discontinuation explaining however only 30% of the variance (*p* < 0.05, R^2^ = 0.31) (S5 Table).

## Discussion

The risk of NTZ-associated PML continuously exposes patients and treating neurologists to difficult treatment decisions. Balancing beneficial effects and risks is a continuing challenge in NTZ treatment [4,24]. The introduction of three-stage risk stratification algorithms has not led to a reduction of PML incidence in NTZ-treated patients. This suggests that factors other than *objective* PML risk play an important role in the highly complex decision-making process concerning therapy initiation and continuation [4,8,20,24–28].

By examining the behavior of patients with different PML risk levels and treating physicians longitudinally in dealing with NTZ therapy in clinical routine and using Bayesian network and regression analyses, we could identify patient- and physician-specific factors significantly related to NTZ treatment cessation. The most striking finding was that the factors “duration of NTZ treatment” and “anti-JCV antibody serostatus”, which define the PML risk of our patients, were not associated with NTZ treatment discontinuation. This means that the *objective* PML risk according to the currently employed risk stratification algorithm [8] probably does not play a dominant role in the patients’ decision to continue or stop NTZ treatment. This finding was surprising and in contrast to previous reports. Thus, it was demonstrated that especially long-term NTZ-treated and JCV Ab + patients are more likely to discontinue NTZ [4,25,29,30]. Tur et al. reported a treatment dropout rate of 23.7% in JCV Ab + patients on NTZ therapy longer than 2 years without prior history of immunosuppression (in contrast to 11% of patients of group C in our study) compared to the 0% in JCV Ab—patients and JCV Ab + patients on NTZ therapy less than 2 years (in contrast to 11% of patients of group A and 20% of patients of group B in our study) [25]. Of note, these treatment dropout rates were evaluated in a cross-sectional study situation where patients had to decide on NTZ continuation/discontinuation after being stratified for PML risk factors, and not within a longitudinal assessment, possibly explaining the discrepancies in our study. Instead, JCV seroconversion during the observation period was significantly associated with discontinuation of therapy (26% (13/50) of patients with JCV seroconversion stopped NTZ). This termination rate was quite high compared to the 11% (15/133) of Patients_stopped NTZ_ of group C. 77% of patients who showed JCV seroconversion (patients switching from group A to B or from group A to C) and stopped NTZ, justified their decision of treatment discontinuation by the high therapeutic risk indicating a lower risk acceptance in patients with initially negligible PML risk (group A). In contrast, only 5% of patients who switched from group B to C during the course of study due to a NTZ treatment duration > 24 months stopped NTZ to change therapy. Tur et al. demonstrated that JCV Ab + patients assumed greater risks than JCV Ab—patients reflecting an adaptive process. Thus, patients who know that they are JCV Ab +, even if they are still on the lower PML risk band, start to assume higher risks earlier than patients who are JCV Ab -, maybe preparing themselves for the near future [26]. Consistent with the result that patients’ assessment of personal PML risk correlated with the proportion of Patients_stopped NTZ_ in JCV Ab + patients and Patients_stopped NTZ_ rated their personal PML risk higher than Patients_Cont. NTZ_, patients’ *subjective* assessment of PML risk significantly influenced their decision to stop NTZ. This result is in line with Heesen et al. who demonstrated that patients were more likely to continue treatment if they perceived the general or personal PML risk as low [31], and explains perhaps why only 11% of patients of group C who assessed their personal PML risk as low, stopped NTZ. Moreover, NTZ treatment cessation was related to physicians’ statement on continuation or discontinuation of therapy. This result is supported by the significant relationship between physicians’ statements on continuation or discontinuation of therapy and the proportion of Patients_stopped NTZ_ and Patients_Cont. NTZ_, and is in line with previous reports demonstrating that especially the views and idiosyncrasies of the treating physician play a prominent role in the decision-making process [8,25]. Interestingly, physicians’ judgments on continuation/discontinuation of therapy depended on the number of patients treated per year suggesting that physicians treating more NTZ-treated MS patients deal differently with PML risk, perhaps due to an increased acceptance and good experience with NTZ, the rarity of PML, and the low rates of AEs and SAEs in daily practice. The missing relationship between NTZ treatment cessation and the physicians’ assessment of PML risk is not surprising because physicians were asked to evaluate the PML risk in general, but not for the individual NTZ-treated patient. A possible explanation for the missing relationship between treatment discontinuation and patients’ risk tolerance and perceived NTZ efficacy could come from the fact that patients were only asked polar questions (yes–no questions) on whether the risk of NTZ (PML) makes them afraid, and if they are prepared to take therapeutic risks to live a “normal” life. The finding that patients’ evaluation of MS severity was not related to treatment discontinuation is in line with recent studies demonstrating that patients’ perception of their own MS as severe disease, but not of MS in general was related to higher risk acceptance [26,31,32].

Compared to the usually described JCV-seroconversion rate of 8–12% per year [17,33,34], our rate of 13% lies in the normal range. However, seroconversion rates of approximately 2–3% per year [8,24,35] but also of 15% [36] and 27% per year [37]. These differences may be due to selection biases.

Our study contains some limitations which have to be pointed out. The average observation period of 264.8 days was relatively short and the proportion of patients with fully completed questionnaires decreased over the duration of the study. This is also the reason why the Bayesian network analysis could only be performed with information from 479 patients of subgroups A-C and the associated 92 neurologists. To achieve high validity, the regression analysis should have been performed as a confirmatory analysis with other patients than those used in the Bayesian network analysis. By excluding patients with immunosuppressive pre-treatment (groups D and E) from statistical analyses, our results are somewhat limited, especially since patients with the potentially highest PML risk (group E: JCV Ab +, IMS +, NTZ > 24 months) could not be sufficiently analyzed. However, since the number of NTZ treated patients with prior use of immunosuppressants is really small in clinical routine, immunosuppressive pre-treatment will not be one of the main reasons for discontinuation of NTZ. Physicians were asked for the reasons why patients stopped NTZ, but not the patients themselves. It would be of particular interest if the reasons given by patients for treatment discontinuation would match the statements of physicians. Moreover, physicians were only requested to evaluate the PML risk in general but not for the individual patient and more importantly, it was not asked if physicians have had experience with patients developing PML. Other factors influencing the decision-making process concerning the continuation of treatment such as patients’ prior experience with DMT, severity of MS, employment status, and social factors [4,8,24–28] were not considered. The influence of patients’ anti-JCV Ab index level on treatment discontinuation could not be evaluated because the study ended in August 2014 and the introduction of levels of anti-JCV Ab (index) to the PML-risk stratification has only been implemented in February 2016 [38].

## Conclusion

Treatment decisions in NTZ therapy seem to be less dependent on patients’ *objective* PML risk according to the PML risk stratification algorithms [8] than previously expected. The decision-making process is rather guided by *subjective* views and experiences of patients and treating neurologists. Therefore, physicians should be aware of the different weighting of *subjective* and *objective* factors in their advice to achieve the best-possible risk-benefit ratio for the individual patient. Because individualized risk prediction is desirable [7], future larger longitudinal studies are necessary to systematically assess each patient´s benefit-risk situation especially of those patients discontinuing NTZ and those continuing NTZ despite high PML risk.

## Supporting information

S1 Fig Patients’ and physicians’ schedule. All visits followed a specific predefined schedule.
(TIFF)

S2 Fig Patients’ evaluation of their actual status of health compared to prior visit (at baseline to a year ago) for patients who continued NTZ (blue) and discontinued NTZ (green) for the visits 1–5. Both patients continuing and discontinuing NTZ showed a slight upward trend in their evaluation of actual status of health during the first three visits. While the assessment of actual status of health further showed a slight improvement in patients continuing NTZ, it worsened at the end of the study in patients discontinuing NTZ.
(TIFF)

S1 Table **Overview over the last received therapies of patients of the groups A-C before starting Natalizumab** (IMS + /- = prior / no prior use of immunosuppressants; JCV Ab + /- = positive / negative serostatus for anti-JCV antibodies; NTZ = natalizumab; NTZ > / ≤ 24 months = NTZ treatment longer / less than 24 months).
(PDF)

S2 Table Overview over the serious adverse events, their severity, outcome, relationship to and impact on natalizumab treatment. A total of 46 serious adverse events were documented for 28 of 669 patients throughout the study period (N = number; NTZ = natalizumab; PML = Progressive multifocal leukoencephalopathy; SAE = serious adverse events).
(PDF)

S3 Table **Patients’ assessment of personal PML risk for patients continuing and discontinuing natalizumab of all subgroups (A-C) for each visit.** Patients discontinuing natalizumab assessed the personal PML risk higher in all visits than patients continuing natalizumab.
(PDF)

S4 Table Physicians’ evaluation of PML risk in general and opinion on continuation/discontinuation of NTZ (mean values for each visit). Physicians assessed the general PML risk as low and mainly voted for treatment continuation on a VAS from 0 to 25 points (SD = standard deviation).
(PDF)

S5 Table Regression analysis with information of 479 patients and treating 92 neurologists. When conducting a regression analysis with information of 479 patients, only patients’ perception of personal PML risk, physicians’ opinion on continuation/discontinuation of NTZ, and the fact if patients showed JCV seroconversion during study or not were significantly related to NTZ discontinuation (R^2^ = 0.31).
(PDF)

S1 Appendix Questionnaires of physicians and patients (English version) (DOCX)

S2 Appendix Questionnaires of physicians and patients (German version) (DOCX)

## Abbreviations

Ab: antibodies
AE: adverse events
ARR: annualized relapse rate
DD: disease duration
DMT: disease modifying therapies
EDSS: Expanded Disability Status Scale
IMS +: prior use of immunosuppressants
JCV: John Cunningham virus
JCV Ab +: positive serostatus for anti-JCV antibodies
JCV Ab -: negative serostatus for anti-JCV antibodies
MS: multiple sclerosis
NTZ: natalizumab
Patients_Cont. NTZ_: patients who continued the therapy with natalizumab over the whole observation period
Patients_Stopped NTZ_: patients who stopped the therapy with natalizumab during the observation period
PML: progressive multifocal leukoencephalopathy
PML_General_: PML risk in general
PML_Personal_: personal PML risk
QoL: quality of life
RRMS: relapsing-remitting multiple sclerosis
SAE: serious adverse events
SD: standard deviation
VAS: visual analog scales
y: years
