# Supplementary figures and images for: Is the risk of progressive multifocal leukoencephalopathy the real reason for natalizumab discontinuation in patients with multiple sclerosis?
Source: PLoS One. 2017 Apr 13;12(4):e0174858. doi: 10.1371/journal.pone.0174858 (PMC5391008; doi:10.1371/journal.pone.0174858)

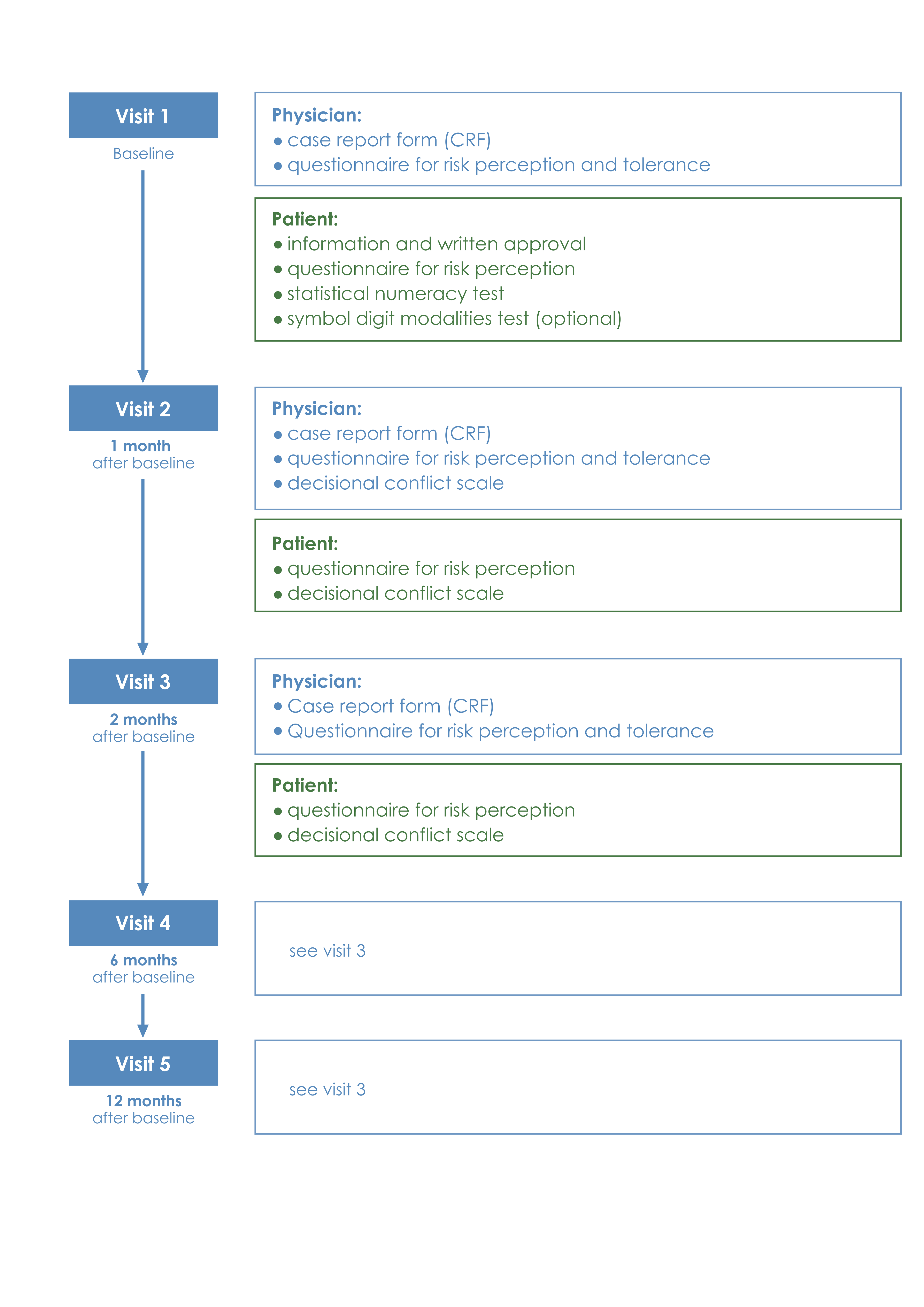

Supplement: S1 Fig — All visits followed a specific predefined schedule. (TIFF) [file pone.0174858.s001.tiff]

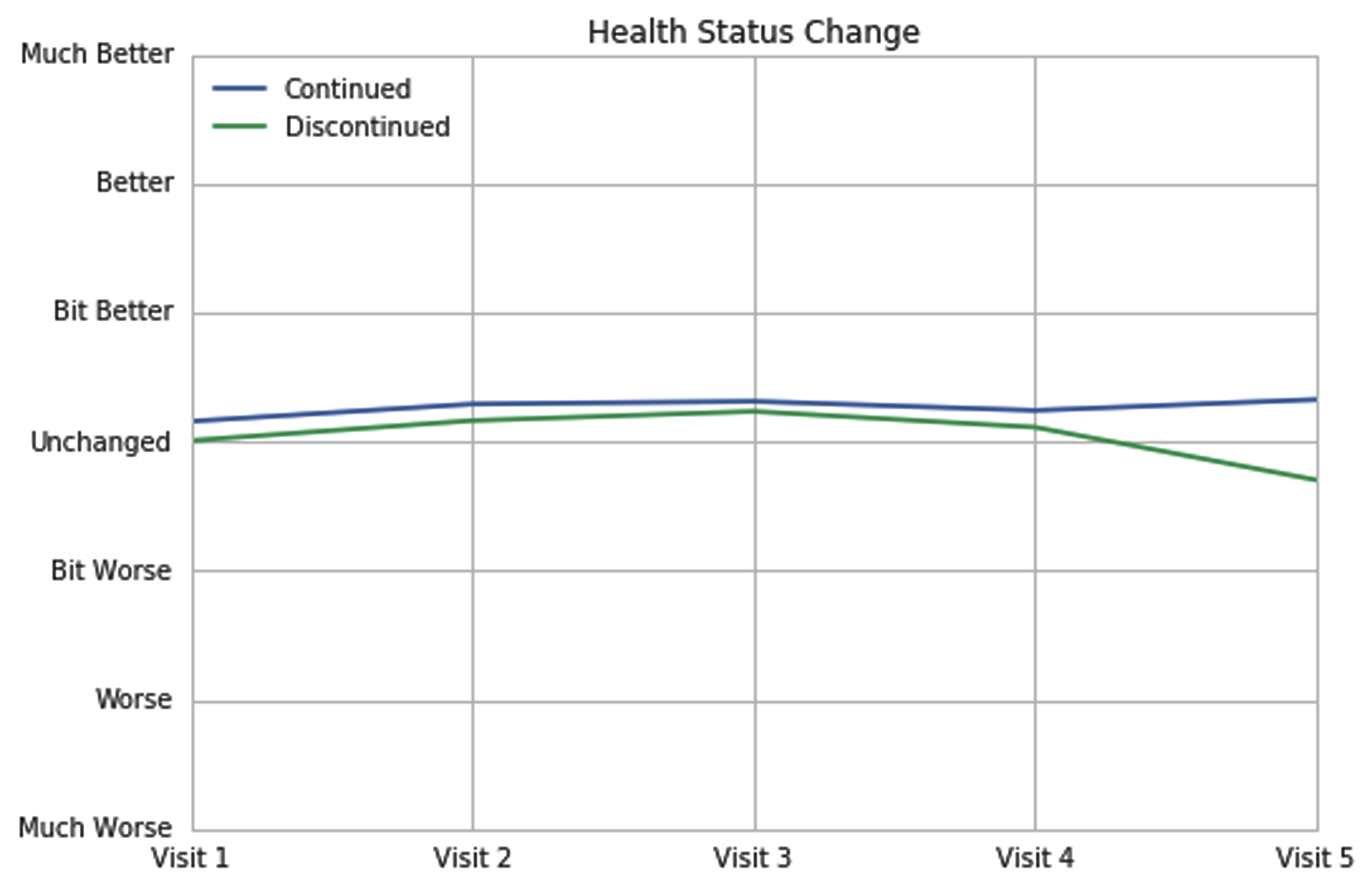

Supplement: S2 Fig — Both patients continuing and discontinuing NTZ showed a slight upward trend in their evaluation of actual status of health during the first three visits. While the assessment of actual status of health further showed a slight improvement in patients continuing NTZ, it worsened at the end of the study in patients discontinuing NTZ. (TIFF) [file pone.0174858.s002.tiff]
