# Supplementary material for: Is the risk of progressive multifocal leukoencephalopathy the real reason for natalizumab discontinuation in patients with multiple sclerosis?
Source: PLoS One. 2017 Apr 13;12(4):e0174858. doi: 10.1371/journal.pone.0174858 (PMC5391008; doi:10.1371/journal.pone.0174858)
Supplement: S1 Table — Overview over the last received therapies of patients of the groups A-C before starting Natalizumab (IMS + /- = prior / no prior use of immunosuppressants; JCV Ab + /- = positive / negative serostatus for anti-JCV antibodies; NTZ = natalizumab; NTZ > / ≤ 24 months = NTZ treatment longer / less than 24 months). (PDF) [file pone.0174858.s003.pdf]

	Group A (JCV Ab –)	Group B (JCV Ab +, IMS -, NTZ ≤ 24 months)	Group C (JCV Ab +, IMS -, NTZ > 24 months)
Azathioprine	4	0	0
Fingolimod	16	14	1
Glatiramer acetate	77	42	35
Immunoglobulin	3	3	2
Interferon beta-1a	154	55	64
Interferon beta-1b	66	26	22
Mitoxantrone	10	0	0
No Therapy	40	20	9
Plasmapheresis	1	0	0
Natalizumab	2	0	0
Steroid pulse therapy	1	0	0
Pegylated interferon beta-1a	0	1	0
Fumaric Acid	0	1	0
