# Supplementary material for: Is the risk of progressive multifocal leukoencephalopathy the real reason for natalizumab discontinuation in patients with multiple sclerosis?
Source: PLoS One. 2017 Apr 13;12(4):e0174858. doi: 10.1371/journal.pone.0174858 (PMC5391008; doi:10.1371/journal.pone.0174858)
Supplement: S2 Table — A total of 46 serious adverse events were documented for 28 of 669 patients throughout the study period (N = number; NTZ = natalizumab; PML = Progressive multifocal leukoencephalopathy; SAE = serious adverse events). (PDF) [file pone.0174858.s004.pdf]

	**Relationship between SAEs and NTZ treatment**	**Severity**	**Outcome**	**Impact on NTZ treament**
**Multiple sclerosis relapses** (N = 14)	unrelated	severe	recovered with sequelae	no action taken
	unrelated	severe	not recovered	stopped temporarily
	unrelated	moderate	recovered	stopped temporarily
	unrelated	moderate	recovered	stopped temporarily
	unrelated	moderate	recovered	withdrawn
	unrelated	moderate	recovered with sequelae	no action taken
	unrelated	mild	recovered	no action taken
	unrelated	mild	recovered with sequelae	stopped permanently
	unrelated	mild	not recovered	stopped temporarily
	unrelated	unknown	recovered	no action taken
	unrelated	unknown	recovered	no action taken
	unrelated	unknown	recovered	no action taken
	unrelated	unknown	recovered with sequelae	no action taken
	unknown	unknown	unknown	stopped temporarily
**Cardiac disorders** (N = 5)	**related**	**severe**	**recovered**	**stopped permanently**
	**related**	**severe**	**recovered**	**stopped permanently**
	unrelated	severe	recovered	stopped permanently
	unrelated	severe	recovered with sequelae	no action taken
	unrelated	severe	recovered with sequelae	no action taken
**Infections** (N = 2)	unrelated	unknown	not recovered	stopped permanently
	unknown	moderate	recovered with sequelae	stopped temporarily
**Neurological disorders** (N = 3)	**related**	**unknown**	**not recovered**	**withdrawn**
	unrelated	mild	not recovered	stopped permanently
	unknown	unknown	recovered	stopped permanently
**PML** (N = 3)	**related**	**severe**	**not recovered**	**stopped permanently**
	**related**	**moderate**	**not recovered**	**stopped permanently**
	**related**	**unknown**	**recovered with sequelae**	**stopped permanently**
**Immune reconstitution inflammatory syndrome** (N = 3)	**related**	**unknown**	**recovered with sequelae**	**stopped permanently**
	**related**	**unknown**	**not recovered**	**stopped permanently**
	unknown	unknown	not recovered	stopped permanently
**Allergic reactions** (N = 2)	**related**	**severe**	**recovered**	**stopped permanently**
	unknown	severe	recovered	stopped permanently
**Neoplasms** (N = 2)	unknown	severe	not recovered	stopped temporarily
	unknown	unknown	recovered	stopped permanently
**Pregnancy complications** (N = 2)	unknown	unknown	recovered	no action taken
	unknown	unknown	unknown	withdrawn
**Progression of multiple sclerosis** (N = 2)	**related**	**severe**	**not recovered**	**withdrawn**
	unrelated	unknown	recovered	no action taken
**Acute renal failure** (N = 1)	unrelated	unknown	not recovered	stopped permanently
**Autonomic dysfunction** (N = 1)	**related**	**moderate**	**recovered**	**stopped permanently**
**Depressive symptom aggravated** (N = 1)	unrelated	moderate	not recovered	no action taken
**Femoral neck fracture**	unrelated	severe	recovered	no action taken

(N = 1)			with sequelae	
**Hypoxic brain damage** (N = 1)	unrelated	severe	fatal	stopped permanently
**Postoperative bleeding** (N = 1)	unrelated	severe	recovered	stopped permanently
**Retinal detachment** (N = 1)	unknown	unknown	recovered with sequelae	no action taken
**Sinus tachycardia** (N = 1)	unrelated	severe	recovered with sequelae	no action taken
