# Supplementary material for: Is the risk of progressive multifocal leukoencephalopathy the real reason for natalizumab discontinuation in patients with multiple sclerosis?
Source: PLoS One. 2017 Apr 13;12(4):e0174858. doi: 10.1371/journal.pone.0174858 (PMC5391008; doi:10.1371/journal.pone.0174858)
Supplement: S3 Table — Patients’ assessment of personal PML risk for patients continuing and discontinuing natalizumab of all subgroups (A-C) for each visit. Patients discontinuing natalizumab assessed the personal PML risk higher in all visits than patients continuing natalizumab. (PDF) [file pone.0174858.s005.pdf]

	Patients continuing NTZ			Patients discontinuing NTZ		
	A	B	C	A	B	C
Visit 1	8.64	12.67	11.40	11.57	13.72	11.53
Visit 2	9.21	13.02	11.92	12.22	12.67	12.37
Visit 3	9.52	14.01	12.37	12.70	14.89	10.89
Visit 4	9.24	13.43	12.70	14.05	15.25	16.73
Visit 5	9.70	13.43	12.95	13.00	14.71	14.47
