# Supplementary material for: Is the risk of progressive multifocal leukoencephalopathy the real reason for natalizumab discontinuation in patients with multiple sclerosis?
Source: PLoS One. 2017 Apr 13;12(4):e0174858. doi: 10.1371/journal.pone.0174858 (PMC5391008; doi:10.1371/journal.pone.0174858)
Supplement: S4 Table — Physicians assessed the general PML risk as low and mainly voted for treatment continuation on a VAS from 0 to 25 points (SD = standard deviation). (PDF) [file pone.0174858.s006.pdf]

	PML risk in general [Mean (SD)]	opinion on continuation/ discontinuation of NTZ [Mean (SD)]
Visit 1	7.80 (5.10)	5.10 (4.02)
Visit 2	8.32 (5.27)	5.08 (3.58)
Visit 3	8.49 (5.08)	5.21 (3.82)
Visit 4	8.83 (4.89)	5.73 (4.95)
Visit 5	7.96 (4.91)	7.19 (6.39)
