# Supplementary material for: Is the risk of progressive multifocal leukoencephalopathy the real reason for natalizumab discontinuation in patients with multiple sclerosis?
Source: PLoS One. 2017 Apr 13;12(4):e0174858. doi: 10.1371/journal.pone.0174858 (PMC5391008; doi:10.1371/journal.pone.0174858)
Supplement: S5 Table — When conducting a regression analysis with information of 479 patients, only patients’ perception of personal PML risk, physicians’ opinion on continuation/discontinuation of NTZ, and the fact if patients showed JCV seroconversion during study or not were significantly related to NTZ discontinuation (R2 = 0.31). (PDF) [file pone.0174858.s007.pdf]

	Standard deviation	z	*p*
Patients' evaluation of personal PML risk	0.04	2.69	0.007
Duration of natalizumab therapy	0.44	-0.84	0.40
Physicians' last opinion on continuation/ discontunation of natalizumab	0.03	6.10	< 0.001
Total number of relapses during the course of study	0.29	0.86	0.39
JCV seroconversion during the course of the study	0.56	2.04	0.04
Number of treated MS patients per year	0.001	-0.52	0.60
Physicians' evaluation of PML risk in general	0.047	1.74	0.08
