# Supplementary material for: Is the risk of progressive multifocal leukoencephalopathy the real reason for natalizumab discontinuation in patients with multiple sclerosis?
Source: PLoS One. 2017 Apr 13;12(4):e0174858. doi: 10.1371/journal.pone.0174858 (PMC5391008; doi:10.1371/journal.pone.0174858)
Supplement: S1 Appendix — (DOCX) [file pone.0174858.s008.docx]

**Appendix: Questionnaires**

**Physician Questionnaire**

1. What is your personal assessment of the benefits of Tysabri in general?

🞎 Helps a lot

🞎 Helps somewhat

🞎 Helps a little

🞎 Does not help

🞎 I don’t know.

Questions about risk perception in MS (Visual Analogue Scale)

*Please tick on the line!*

2. Please provide an assessment:

MS is a rather **benign** disease - MS is a rather **severe** disease

3. Please provide an assessment for the present patient:

MS is a rather **benign** disease - MS is a rather **severe** disease

Questions about PML risk tolerance (rf. Heesen 2010) (Visual Analogue Scale)

*Please tick on the line!*

4. Do you GENERALLY assess the risk of developing PML as high or low?

Low - High

5. Based on this updated information, would you rather continue or discontinue treating the patient with Tysabri?

*Please tick on the line!*

More likely to continue – More likely to discontinue

**Patient Questionnaire**

Clinical Global Impression

1. Compared to the situation one year ago (in case of follow-up visits, compared to the previous visit), how would you describe your status of health?

🞎 Considerably worse

🞎 Noticeably worse

🞎 Minimally worse

🞎 No change

🞎 Minimally improved

🞎 Noticeably improved

🞎 Considerably improved

2. I am currently satisfied with my quality of life.

🞎 Not at all

🞎 Somewhat satisfied

🞎 Moderately satisfied

🞎 Quite satisfied

🞎 Very satisfied

Questions about the personal decision for treatment with Tysabri

3. Which aspects were important for the decision for Tysabri?
Other medications had no effect. 🞎 Yes 🞎 No

Tysabri is a very effective medication. 🞎 Yes 🞎 No

I was in a desperate situation. 🞎 Yes 🞎 No

There were no alternatives. 🞎 Yes 🞎 No

I was prepared to risk something in order to lead a normal

life again. 🞎 Yes 🞎 No

I’m not afraid of the risks (PML). 🞎 Yes 🞎 No

Other reasons:……………………………………………………

Questions about risk perception in MS (rf. Boeije 2004, Heesen 2010) (Visual Analogue Scale)

*Please tick on the line!*

4. Please provide an assessment:

MS is a rather **benign** disease - MS is a rather **severe** disease

5. What would dependence on a wheelchair imply for you? (rf. Boeije 2004)

It wouldn’t matter much – It’s the worst thing I can imagine

Questions about PML risk tolerance

6. How would you GENERALLY rate the risk of developing PML?

Low - High

7. Based on your MS history and considering your handling of risks generally: How would you rate YOUR OWN risk of PML compared to other patients in the same situation?

Lower - Higher
