# Supplementary material for: Is the risk of progressive multifocal leukoencephalopathy the real reason for natalizumab discontinuation in patients with multiple sclerosis?
Source: PLoS One. 2017 Apr 13;12(4):e0174858. doi: 10.1371/journal.pone.0174858 (PMC5391008; doi:10.1371/journal.pone.0174858)
Supplement: S2 Appendix — (DOCX) [file pone.0174858.s009.docx]

**Anhang: Fragebögen**

**Fragebögen Ärzte**

1. Wie ist Ihre persönliche Einschätzung des Nutzens von Tysabri im Allgemeinen?

🞎 Hilft sehr

🞎 Hilft etwas

🞎 Hilft wenig

🞎 Hilft eher nicht

🞎 Ich weiß nicht

Fragen zur Risikowahrnehmung bei MS (Visuelle Analogskala)

*Bitte auf der Linie ankreuzen!*

2. Bitte schätzen Sie ein:

MS ist eine eher **gutartige** Erkrankung - MS ist eine eher **schwere** Erkrankung

3. Bitte schätzen Sie für den vorliegenden Patienten ein:

MS ist eine eher **gutartige** Erkrankung - MS ist eine eher **schwere** Erkrankung

Fragen zur PML-Risikotoleranz (vgl. Heesen 2010) (Visuelle Analogskala)

*Bitte auf der Linie ankreuzen!*

4. Schätzen Sie das PML-Erkrankungsrisiko GENERELL als hoch oder gering ein?

Gering - Hoch

5. Werden Sie mit diesen aktuellen Informationen die Tysabri-Therapie bei dem vorliegenden Patienten eher fortsetzen oder abbrechen?

*Bitte auf der Linie ankreuzen!*

Eher fortsetzen – Eher abbrechen

**Fragebögen Patienten**

Clinical Global Impression

1. Im Vergleich zu der Situation vor einem Jahr (bei Folgevisiten im Vergleich zur letzten Visite): Wie würden Sie Ihre Gesundheitssituation beschreiben?

🞎 Massiv schlechter

🞎 Deutlich schlechter

🞎 Etwas schlechter

🞎 Gleich

🞎 Etwas besser

🞎 Deutlich besser

🞎 Erheblich besser

2. Ich bin derzeit mit meiner Lebensqualität zufrieden.

🞎 Gar nicht

🞎 Ein wenig

🞎 Mäßig

🞎 Ziemlich

🞎 Sehr

Fragen zur persönlichen Therapieentscheidung für Tysabri

3. Welche Aspekte waren für die Tysabri Entscheidung wichtig?
Andere Medikamente haben nicht geholfen. 🞎 Ja 🞎 Nein

Tysabri ist ein sehr wirksames Medikament. 🞎 Ja 🞎 Nein

Ich war in einer ausweglosen Lage. 🞎 Ja 🞎 Nein

Es gab keine Alternativen. 🞎 Ja 🞎 Nein

Ich war bereit etwas zu riskieren, um wieder ein normales

Leben führen zu können. 🞎 Ja 🞎 Nein

Die Risiken (PML) machen mir keine Angst. 🞎 Ja 🞎 Nein

Andere Gründe: ……………………………………………………

Fragen zur Risikowahrnehmung bei MS (vgl. Boeije 2004, Heesen 2010) (Visuelle Analogskala)

*Bitte auf der Linie ankreuzen.*

4. Bitte schätzen Sie ein:

MS ist eine eher **gutartige** Erkrankung - MS ist eine eher **schwere** Erkrankung

5. Was würde eine Rollstuhlabhängigkeit für Sie bedeuten? (vgl. Boeije 2004)

Wär für mich gar nicht schlimm – Das Schlimmste, das ich mir vorstellen kann

Fragen zur PML-Risikotoleranz

6. Wie schätzen Sie das PML-Erkrankungsrisiko GENERELL ein?

Gering - Hoch

7. Vor dem Hintergrund Ihrer MS-Krankengeschichte und mit Berücksichtigung Ihres Umgangs mit Risiken generell: Wie schätzen Sie das Risiko für eine PML für sich PERSÖNLICH im Vergleich zu anderen Patienten in der gleichen Situation ein?

Geringer - Höher
